# High incidence of rainbow glare after femtosecond laser assisted-LASIK using the upgraded FS200 femtosecond laser

**DOI:** 10.1186/s12886-018-0734-1

**Published:** 2018-03-05

**Authors:** Yu Zhang, Yue-guo Chen

**Affiliations:** Department of Ophthalmology, Peking University Third Hospital, 49 North Huayuan Road, Haidian District, Beijing, 100191 China

**Keywords:** Rainbow glare, Incidence, Fs-LASIK, FS200 femtosecond laser

## Abstract

**Background:**

To compare the incidence of rainbow glare (RG) after femtosecond laser assisted-LASIK (FS-LASIK) using the upgraded FS200 femtosecond laser with different flap cut parameter settings.

**Methods:**

A consecutive series of 129 patients (255 eyes) who underwent FS-LASIK for correcting myopia and/or astigmatism using upgraded WaveLight FS200 femtosecond laser with the original settings was included in group A. Another consecutive series of 129 patients (255 eyes) who underwent FS-LASIK using upgraded WaveLight FS200 femtosecond laser with flap cut parameter settings changed (decreased pulse energy, spot and line separation) was included in group B. The incidence and fading time of RG, confocal microscopic image and postoperative clinical results were compared between the two groups.

**Results:**

There were no differences between the two groups in age, baseline refraction, excimer laser ablation depth, postoperative uncorrected visual acuity and refraction. The incidence rate of RG in group A (35/255, 13.73%) was significantly higher than that in group B (4/255, 1.57%) (*P* < 0.05). The median fading time was 3 months in group A and 1 month in group B (*P* > 0.05).The confocal microscopic images showed wider laser spot spacing in group A than group B. The incidence of RG was significantly correlated with age and grouping (*P* < 0.05).

**Conclusions:**

The upgraded FS200 femtosecond laser with original flap cut parameter settings could increase the incidence of RG. The narrower grating size and lower pulse energy could ameliorate this side effect.

## Background

Femtosecond laser-assisted flap creation in LASIK surgery has rapidly become popular with increasing safety and efficacy over time [1–4]. When using femtosecond laser for flap creation, each pulse of the laser causes the generation of a small amount of microplasma at its focal point in the corneal tissue leading to formation of microscopic gas bubbles and cavitations, which then dissipate into surrounding tissue [5, 6]. Compared with mechanical microkeratome for making flaps, a key benefit of femtosecond laser technology is to provide a more precision, predictable flap and less vision threatening flap complications [7, 8]. However, the femtosecond laser technique, as the resulting laser pattern may act as an optical grating, could be a major drawback. Previous studies indeed reported on perceptions of rainbow glare (RG) as a mild side effect of FS-LASIK and was verified on a model eye [9–11].

WaveLight FS200 femtosecond laser system (Alcon Laboratories Inc., Fort Worth, TX) is a high pulse frequency (repetition rate of 200 kHz) and comparatively lower pulse energy system that can produce flaps in a shorter period of time, around 6 s. Flaps created using FS200 demonstrated both precision and reproducibility with minimal side effects [12–14]. Only two previous studies reported two cases of unilateral RG following FS-LASIK with the WaveLight FS-200 femtosecond laser [15, 16]. To our knowledge, there is no published study on incidence rate and alleviating management of RG when using FS200 femtosecond laser. In this retrospective study, we reported a high incidence of RG after FS-LASIK using the upgraded FS200 and evaluated the alleviative effect of adjustment of flap cut parameter settings.

## Methods

### Subjects

This was a retrospective cohort study conducted at Peking University Third Hospital from December 2015 to May 2017. It was carried out in accordance with the tenets of the Declaration of Helsinki and approved by the Ethics Committee of Peking University Third Hospital. An informed consent was obtained from each subject.

A consecutive series of 129 patients (255 eyes) who underwent FS-LASIK for correcting myopia and/or myopic astigmatism using upgraded WaveLight FS200 femtosecond laser with original flap cut parameter settings was included in group A. Another consecutive series of 129 patients (255 eyes) who in the period immediately following group A underwent FS-LASIK using same laser platform with flap cut parameter setting changed was included in group B.

### Surgical procedures

**Fig. 1:**
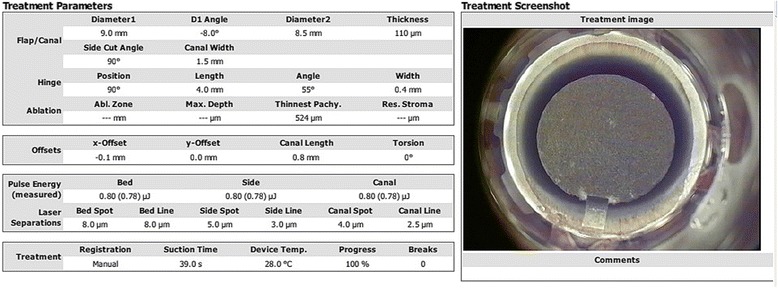
Energy, bed spot and line parameters used for group A and one example of snapshot taken right after the completion of the interface creation

**Fig. 2:**
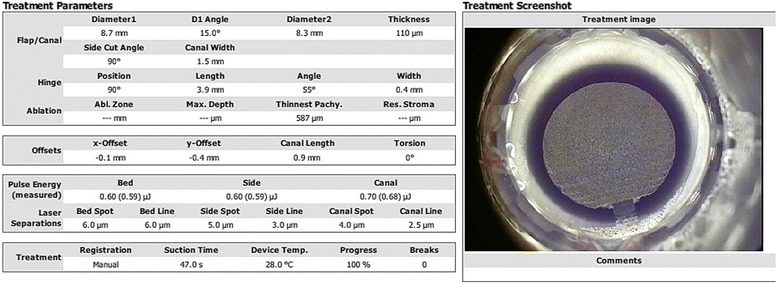
Energy, bed spot and line parameters used for group B and one example of snapshot taken right after the completion of the interface creation

All surgical procedures were performed by the same surgeon (Yue-guo Chen). All flaps were created by the WaveLight FS200 laser. The flap/canal/hinge parameters were as followed: flap thickness, 110 μm; flap diameter, 8.5 mm to 9.0 mm; side-cut angle, 90°; hinge angle, 50°; canal width, 1.5 mm. For group A, after FS200 laser was upgraded, the energy and laser separations settings were the same to the original one: side-cut pulse energy, 0.8 μJ; bed cut pulse energy, 0.8 μJ; stromal bed cut spot separation, 8 μm; line separation, 8 μm; side cut bed separation, 5 μm; and line separation, 3 μm (Fig. 1). For group B, all settings was the same to group A except for side-cut and bed cut pulse energy, 0.6 μJ; stromal bed cut spot separation and line separation, 6 μm (Fig. 2). Every flap was superiorly hinged, with a superior gas canal. Flaps were lifted immediately after flap creation to perform ablation using the Allegretto EX500 excimer laser (Alcon Laboratories Inc., Fort Worth, TX).

### Postoperative care and follow-up

The standard postoperative regimen included one drop each of 0.5% levofloxacin (Cravit, Santen, Inc.) and 0.1% fluorometholone (FML, Allergan, Inc.) QID for 2 weeks. Each patient was followed up at days 1 and 7, and months 1, 3, 6 and 12 after surgery. The follow-up examinations involved measurements of uncorrected visual acuity (UCVA), slit-lamp examination, subjective refraction, best corrected visual acuity, corneal topography (Sirius, CSO, Italy), and HRT II confocal microscope (Heidelberg Engineering, GmbH, Dossenheim, Germany). The acquired two- dimensional image by HRT II is defined by 384 × 384 pixels covering an area of 400 × 400 μm with lateral digital resolution of 1 μm/pixel and digital depth resolution of 2 μm/pixel.

### Determination of rainbow glare symptom

During follow-up, the patients complained seeing a spectrum of colored bands radiating from a white-light source when viewed in a dark environment and could match the sample pictures of rainbow glare described by Krueger RR et al [9]. Then determination of the presence of RG was made.

### Statistical analysis

Data were analyzed using SPSS 21.0 (SPSS Inc., Chicago, Illinois). Independent-samples *t* test (normal distribution), independent-samples Mann-Whitney U test (non-normal distribution), Pearson Chi-Square test and Kaplan-Meier analysis were used to compare data between the two groups. Spearman bivariate correlation was used to analyze the correlative factors of rainbow glare. A *P* value < 0.05 was considered statistically significant.

## Results

### Visual acuity and refractive results

There were no significant differences between the two groups in age, preoperative refraction, and excimer laser ablation depth (Table 1). At postoperative 1, 3, 12-month, there were no significant differences in UCVA and spherical equivalence (SE) between the two groups (*P* > 0.05).

**Table 1:** Refraction and demographic data of the two groups ($\overline{x}$±s/ M)

	Group A	Group B	t / Z	*P*
Age (years)	27.62±6.25	26.24±7.02	1.597	0.111
SE(D)	−6.36±2.04	−6.47±2.26	0.617	0.535
Cyl(D)	−0.75 (0~ − 3.25)	−0.75 (0~ − 4.25)	2.143	0.062
AD (μm)	92.06±21.02	94.71±22.20	−1.43	0.153

*SE* spherical equivalent, *Cyl* cylinder, *AD* excimer laser ablation depth

### Incidence of rainbow glare

**Fig. 3:**
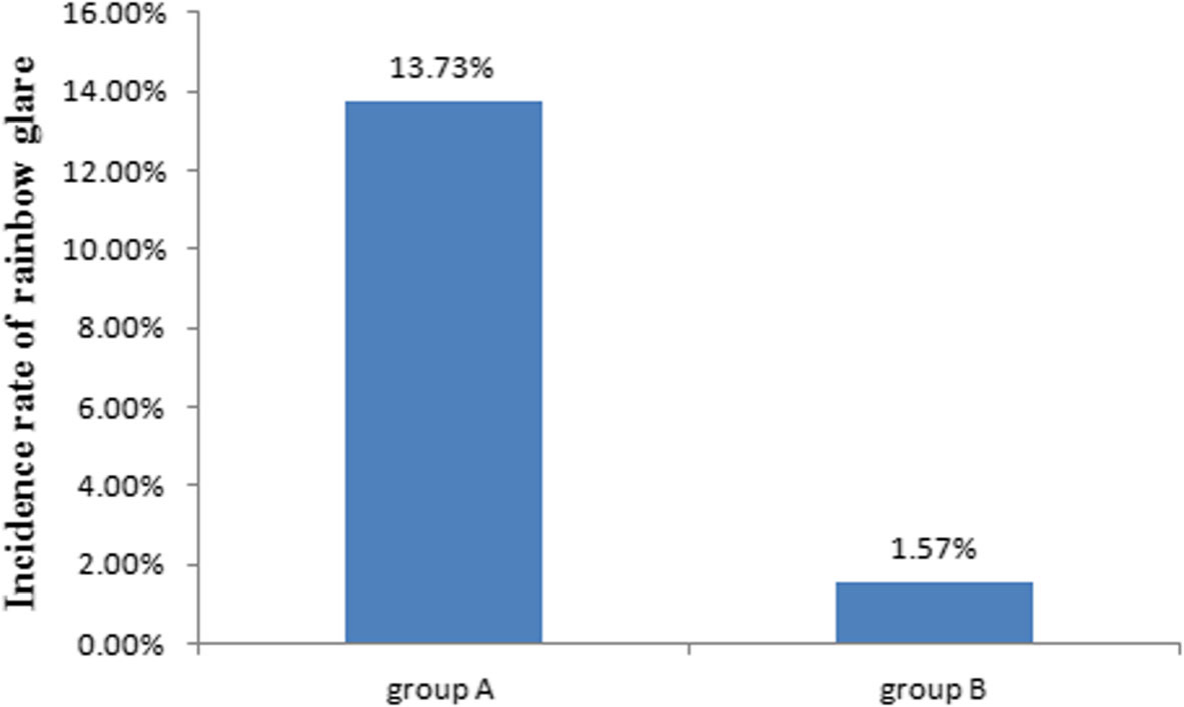
Incidence rate of rainbow glare in the two groups

Eighteen patients (35 eyes: 17 bilateral; 1 unilateral) complained RG in group A. However, only two patients (4 eyes) reported RG in group B. The difference in the incidence rate of rainbow glare between the two groups was statistically significant (*P* < 0.05) (Fig. 3). The symptom arose within one week among most of the patients (18/20, 90%).

### Fading time of rainbow glare

**Fig. 4:**
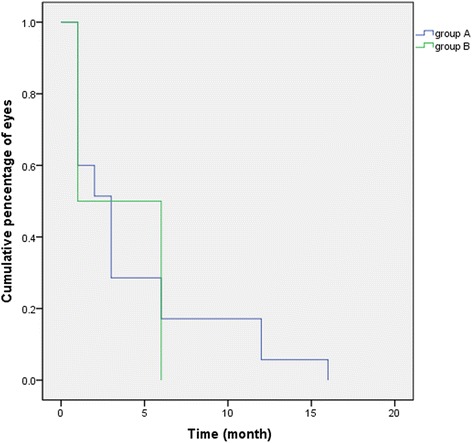
The fading time of rainbow glare in the two groups

Rainbow glare faded away as time went by. In group A, all the patients experienced complete resolution of symptom by the 16th postoperative month. In group B, rainbow glare faded away by the 6th postoperative month (Fig. 4). The median fading time was 3 months in group A and 1 month in group B. However, there was no significant difference in fading time between the two groups (Log Rank, χ^2^ = 0.044, *P* = 0.834).

### Confocal microscopic results

**Fig. 5:**
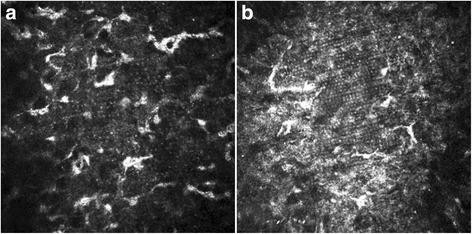
Three months post-operation, HRT II corneal confocal microscopy showed comparatively wider laser spot spacing in a group A patient (**a**) and narrower spot spacing in a group B patient (**b**) (original magnification 400 × 400 μm)

HRT II confocal microscope equipped with the Rostock corneal module showed the femtosecond laser spots, spot spacing and grating pattern clearly at approximately 100~ 110 μm level below the anterior corneal surface. The images showed comparatively wider laser spot spacing in a patient of group A (Fig. 5a) and narrower spot spacing in a patient of group B (Fig. 5b). Although much more undistinguishable with time, this phenomenon could be found even at 12 months after surgery.

### Comparison between eyes with RG and eyes without RG

Among all the 510 eyes, no significant differences in refraction, UCVA and ablation depth were found between eyes with RG (39 eyes) and eyes without RG (471 eyes). However, the mean age of subjects with RG was significantly older than that of subjects without RG (*P* < 0.05). (Table 2).

**Table 2:** Comparison between eyes with rainbow glare and eyes without rainbow glare ($\overline{x}$±s / M)

	With RG (39 eyes)	Without RG (471 eyes)	t / Z	*P*
Age (years)	29.97±5.01	26.75±6.76	2.584	0.013
Baseline SE (D)	−6.34±2.13	− 6.42±2.16	0.232	0.816
Baseline Cyl (D)	−0.75 (0~ − 3.50)	−0.75 (0~ − 4.25)	0.113	0.984
AD (μm)	90.59±22.96	93.57±21.53	−0.827	0.409
1 M UCVA(LogMAR)	−0.04(− 0.15~ 0.15)	−0.04(− 0.18~ 0.22)	0.206	0.955
1 M SE (D)	0.00 (0.00~ 1.00)	0.13 (− 1.00~ 1.00)	0.198	0.961

*SE* spherical equivalent, *Cyl* cylinder, *AD* excimer laser ablation depth, *RG* rainbow glare, *1 M UCVA* uncorrected visual acuity at postoperative 1 month

### Correlative factors of rainbow glare

In all the 510 eyes, the occurrence of RG was significantly correlated with age (*r* = 0.115, *P* < 0.05) and grouping (*r* = 0.229, *P* < 0.001), but not with baseline SE, baseline cylinder or excimer laser ablation depth (*P* > 0.05).

## Discussion

LASIK is the main stream refractive surgery procedure for correction of myopia and myopic astigmatism worldwide. The introduction of the femtosecond laser in LASIK enabled the creation of lamellar corneal flaps in a more accurate, more stable, evener and safer manner. Even though RG which was first reported in 2008 by Krueger et al. is a mild and temporary optical side effect of FS-LASIK [9], it may disturb patients’ night driving and decrease their satisfaction [9, 10]. The cause of RG was defined as the diffraction of light from the grating pattern created on the back surface of the LASIK flap after femtosecond laser exposure [17]. The first clinical study describing RG found an incidence rate of 19.07% with an older high pulse energy, low frequency (15 kHz) IntraLase femtosecond laser model [9]. A subsequent study reported an incidence of rainbow glare of 5.8% with a newer generation of lower pulse energy, higher frequency (60 kHz) IntraLase model [10].

Since low pulse energy and high frequency femtosecond laser system was applied, less incidence of RG has been reported. The first case of RG with WaveLight FS200 femtosecond laser platform, the lower pulse energy and higher frequency system, was reported by Gatinel D, et al. in 2013 [15]. We have used the WaveLight FS200 femtosecond laser platform (≤0.8 μJ pulse energy for flap, 200 kHz frequency) for performing FS-LASIK since 2011 with the recommended optimized cut settings of 0.80 μJ pulse energy, 8.00 μm spot and line separations for stromal bed cut and barely heard complaining of the RG symptom until the laser platform was upgraded to “Green” version in December, 2015. As we understood, “Green” brought only new features of software, such as patient data management, docking guidance system and flap position registration on the basis of pupil center for more convenient without changing any laser parameter settings. After the high incidence of RG was noted, we got a new recommendation of optimized cut settings of 0.60 μJ pulse energy, 6.00 μm spot and line separations, the tolerable arrangement of former version, for stromal bed cut. Since then, the incidence rate of RG had decreased to 1.57% among the following consecutive series cases.

Up to date, the reason why the incidence of RG significantly increased after machine being upgraded has still been unclear. The quality of the focused laser beam and numerical aperture of the focusing optics appears to be the most significant factor in minimizing the diffractive dispersion of light that leads to RG [9, 10]. Otherwise, the relationship between length of time between service calls and the incidence of RG emphasized the importance of a proper maintenance and alignment of the optical components used to focus the beam [10].

Gatinel D, et al. calculated a grating pattern spacing of 7.90μm was the cause of rainbow glare [15]. This value was very close to 8.0μm in group A of the present study. It might seem that reducing the spot/line separation could increase the angle of the spectral pattern sufficient enough to avoid photodetection by the retina altogether [17]. Correlative results in the present study showed that less laser pulse energy and narrower spot/line separations might play an important role in decreasing the incidence of RG. With the settings changed, the energy intensity and the flap cutting time (from 6 to 7 s to 12–13 s) increased, which may induce more corneal lamellar tissue reaction and should be observed for longer time.

With intensively in vivo confocal microscope scan, at around the 110 μm depth level from the anterior corneal surface of almost all the cases after FS-LASIK in this study, no matter whether RG existed or not, the hyper-reflective spots could be found in an equidistant grid-like pattern with HRT II confocal microscope. The spot pattern matched the laser spots/lines separation of 8.0μm or 6.0μm programmed in the FS200 femtosecond laser. There was a report of successful RG correction using undersurface ablation of the LASIK flap [16]. We considered that it was not necessary to do so if merely the symptom of RG existed, because the symptom of RG disappeared with time in all the subjects of the present study, even when the hyper-reflective spots were still visible under confocal microscopy. In this aspect, the patients’ individual variation of sensitivity and self-adaption might play a pivotal role.

Krueger et al. found an increased degree of myopic correction was associated with an increased incidence of RG [9]. However, the present study did not found correlation between initial SE or ablation depth and the incidence of RG, which was consistent with the study of Bamba et al [10]. The present study first found that older age was a correlative factor of RG. Only one previous report studied the relationship between age and the incidence of RG and found negative result [10]. Different race of subjects and femtosecond laser machine might lead to the reverse results. RG was subjective feeling, and older subjects might have more need in night vision, such as driving in the night, which might cause more older subjects felt and reported RG to the doctor. More prospective large-sample studies are needed in future to explore the risk or correlative factors of RG.

## Conclusions

In conclusion, the upgrade of WaveLight FS200 femtosecond laser with original flap cut parameter settings could increase the incidence of RG. After the pulse energy, spot/line separations for stromal bed cut was adjusted, the incidence of RG decreased significantly. RG did not influence postoperative visual acuity or refractive results. It faded away as time went by. Narrower grating size and lower pulse energy could significantly ameliorate this side effect after FS-LASIK when using WaveLight FS200 femtosecond laser.

## Abbreviations

FS-LASIK: femtosecond laser assisted-laser in situ keratomileusis
RG: rainbow glare
SE: spherical equivalence
UCVA: uncorrected visual acuity

## References

[1] Salomao MQ, Wilson SE (2010). Femtosecond laser in laser in situ keratomileusis. J Cataract Refract Surg.

[2] Farjo AA, Sugar A, Schallorn SC, Majmudar PA, Tanzer DJ, Trattler WB, et al. Femtosecond lasers for LASIK flap creation: a report by the American Academy of ophthalmology. Ophthalmology. 2013;120:e5-e20. 10.1016/j.ophtha.2012.08.013 23174396

[3] Lubatschowski H (2008). Overview of commercially available femtosecond lasers in refractive surgery. J Refract Surg.

[4] Kymionis GD, Kontadakis GA, Naoumidi I, Kankariya VP, Panagopoulou S, Manousaki A (2014). Comparative study of stromal bed of LASIK flaps created with femtosecond lasers (IntraLase FS150, WaveLight FS200) and mechanical microkeratome. Br J Ophthalmol.

[5] Chung SH, Mazur E (2009). Surgical applications of femtosecond laser. J Biophotonics.

[6] Kurtz RM, Liu X, Elner VM, Squier JA (1997). Du D, Mourou GA. Photodisruption in the human cornea as a function of laser pulse width. J Refract Surg.

[7] Reinstein DZ, Archer TJ, Gobbe M, Johnson N (2010). Accuracy and reproducibility of Artemis central flap thickness and visual outcomes of LASIK with the Carl Zeiss Meditec VisuMax femtosecond laser and MEL80 excimer laser platforms. J Refract Surg.

[8] Zhang Y, Chen YG, Xia YJ (2013). Comparison of corneal flap morphology using AS-OCT in LASIK with the WaveLight FS200 femtosecond laser versus a mechanical microkeratome. J Refract Surg.

[9] Krueger RR, Thornton IL, Xu M, Bor Z, van den Berg TJ (2008). Rainbow glare as an optical side effect of IntraLASIK. Ophthalmology.

[10] Bamba S, Rocha KM, Ramos-Esteban JC, Krueger RR (2009). Incidence of rainbow glare after laser in situ keratomileusis flap creation with a 60 kHz femtosecond laser. J Cataract Refract Surg.

[11] Kamm A, Tünnermann A, Merker M, Kamm A, Tünnermann A, Nolte S (2013). Optical side-effects of fs-laser treatment in refractive surgery investigated by means of a model eye. Biomedical Optics Express.

[12] Mochen M, Wüllner C, Krause J, Klafke M, Donitzky C, Seiler T (2010). Technical aspects of the WaveLight FS200 femtosecond laser. J Refract Surg.

[13] Kanellopoulos AJ, Asimellis G (2013). Refractive and keratometric stability in high myopic LASIK with high-frequency femtosecond and excimer lasers. J Refract Surg.

[14] Kanellopoulos AJ, Asimellis G (2013). Digital analysis of flap parameter accuracy and objective assessment of opaque bubble layer in femtosecond laser-assisted LASIK: a novel technique. Clin Ophthalmol.

[15] Gatinel D, Saad A, Guilbert E, Rouger H (2013). Unilateral rainbow glare after uncomplicated Femto-LASIK using the FS-200 femtosecond laser. J Refract Surg.

[16] Gatinel D, Saad A, Guilbert E, Rouger H (2015). Simultaneous correction of unilateral rainbow glare and residual astigmatism by undersurface flap photoablation after femtosecond laser-assisted LASIK. J Refract Surg.

[17] Moshirfar M, Desautels JD, Quist TS, Skanchy DF, Williams MT, Wallace RT (2016). Rainbow glare after laser-assisted in situ keratomileusis: a review of literature. Clin Ophthalmol.

